# Observation versus treatment among men with favorable risk prostate cancer in a community-based integrated health care system: a retrospective cohort study

**DOI:** 10.1186/s12894-018-0372-1

**Published:** 2018-06-04

**Authors:** Furaha Kariburyo, Yuexi Wang, I-Ning ( Elaine) Cheng, Lisa Wang, David Morgenstern, Lin Xie, Eric Meadows, John Danella, Michael L. Cher

**Affiliations:** 1 STATinMED Research, 211 N. Fourth Avenue, Suite 2B, Ann Arbor, MI 48104 USA; 2 Diagnostics Information Solutions, F. Hoffmann-La Roche AG, Basel, Switzerland; 3 Genentech, Inc, South San Francisco, CA USA; 4 Roche Diagnostics Operations, Indianapolis, IN USA; 5 MedMining, Danville, PA USA; 6 Geisinger Health System, Danville, PA USA; 7 Wayne State University School of Medicine and the Barbara Ann Karmanos Cancer Institute, Detroit, MI USA

**Keywords:** Prostate cancer, Active surveillance, Overall survival, Monitoring patterns

## Abstract

**Background:**

The objective of this study was to describe overall survival and the management of men with favorable risk prostate cancer (PCa) within a large community-based health care system in the United States.

**Methods:**

A retrospective cohort study was conducted using linked electronic health records from men aged ≥40 years with favorable risk PCa (T1 or 2, PSA ≤15, Gleason ≤7 [3 + 4]) diagnosed between January 2005 and October 2013. Cohorts were defined as receiving any treatment (IMT) or no treatment (OBS) within 6 months after index PCa diagnosis. Cohorts’ characteristics were compared between OBS and IMT; monitoring patterns were reported for OBS within the first 18 and 24 months. Cox Proportional Hazards models were used for multivariate analysis of overall survival.

**Results:**

A total of 1425 men met the inclusion criteria (OBS 362; IMT 1063). The proportion of men managed with OBS increased from 20% (2005) to 35% (2013). The OBS group was older (65.6 vs 62.8 years, *p* < 0.01), had higher Charlson comorbidity index scores (CCI ≥2, 21.5% vs 12.2%, *p* < 0.01), and had a higher proportion of low-risk PCa (65.2% vs 55.0%, *p* < 0.01). For the OBS cohort, 181 of the men (50%) eventually received treatment. Among those remaining on OBS for ≥24 months (*N* = 166), 88.6% had ≥1 follow-up PSA test and 26.5% received ≥1 follow-up biopsy within the 24 months. The unadjusted mortality rate was higher for OBS compared with IMT (2.7 vs 1.3/100 person-years [py]; *p* < 0.001). After multivariate adjustment, there was no significant difference in all-cause mortality between OBS and IMT groups (HR 0.73, *p* = 0.138).

**Conclusions:**

Use of OBS management increased over the 10-year study period. Men in the OBS cohort had a higher proportion of low-risk PCa. No differences were observed in overall survival between the two groups after adjustment of covariates. These data provide insights into how favorable risk PCa was managed in a community setting.

## Background

The widespread adoption of prostate-specific antigen (PSA)-based screening has led to a substantial increase in the detection of favorable risk prostate cancer (PCa) [1]. PSA-based screening has been shown to reduce prostate-specific mortality by 21–30% [2, 3]. However, the use of PSA screening has resulted in considerable over-diagnosis and over-treatment, with 15–20% of men receiving a PCa diagnosis during their lifetime but only 3% dying from the disease [4].

Observation strategies (OBS) such as active surveillance (AS) and watchful waiting (WW) are alternatives to immediate treatment (IMT) for men diagnosed with favorable risk PCa. The primary motivation for these strategies is to avoid or delay treatment-related adverse events [5].

AS strategies also reduce immediate health care expenditures by avoiding aggressive treatment costs, including treatment complications, but they imply recurrent costs for biopsies and other tests that accumulate over the patient’s lifetime [5]. The risk vs benefit consideration of AS also includes the possibility of disease progression to the point that a cure is less likely or not possible.

Recent studies indicate increased adoption of AS. However, most published reports are from academic centers [6] or prospective trials with defined AS protocols. Some studies have examined updated observational strategies, finding that those who chose to undergo OBS tended to be older with lower risk disease. The number of men diagnosed with low-risk PCa who chose AS in these studies was low, suggesting that AS may have been underused in the management of very low-risk PCa [7, 8]. The objectives of this study were to compare overall survival and the clinical characteristics and management trends (OBS vs IMT) among men with favorable risk PCa from a large community practice, as well as to identify factors associated with choosing OBS and to describe monitoring patterns used for OBS.

## Methods

### Data source

A retrospective study was conducted from January 2004 to April 2015 using linked electronic medical records (EMRs), oncology registry data, and enrollment information from the Geisinger Health System (GHS) – a community-based integrated health care organization serving residents in central, south, and northeast Pennsylvania. The EMR infrastructure contains longitudinal clinical patient data including patient demographics and encounter details from inpatient, outpatient, and office-based settings such as diagnoses, medications orders, procedures, and laboratory results.

### Patient identification

Men aged ≥40 years and diagnosed with favorable risk PCa (International Classification of Diseases [ICD]-O3 site code C61.9 and morphology 81,403, T1 or 2, PSA ≤15 ng/mL, Gleason score ≤ 7 [3 + 4]) identified from January 2005–October 2013 and active in the GHS ≥12 months prior to and ≥ 18 months post-index date were selected*.* The first PCa diagnosis date was defined as the index date. Patient data were assessed until the earlier of death or April 2015.

Patients with evidence of a previous cancer diagnosis ≤5 years prior to the initial PCa diagnosis (except for non-melanoma skin cancer), any PCa treatment before the index PCa diagnosis, or other cancer diagnosis within 6 months after the initial PCa diagnosis (identified using ICD-O3-codes) were excluded.

PCa and other cancer diagnoses, Gleason score, and tumor stage were extracted from the oncology registry. Demographic, encounter, and PSA testing information were retrieved from EMR data. Cohorts were defined as receiving any PCa treatment (IMT) or no treatment within 6 months (OBS) after index PCa diagnosis date.

Prostate cancer risk categories were defined by D’Amico classification [9]: low (T1-T2A, PSA level ≤ 10 ng/mL, and Gleason score ≤ 6) [10] or intermediate (T1 or T2, PSA level > 10 and ≤ 20 ng/mL, and Gleason score = 7).

### IRB approval

All patient information was de-identified at the source in accordance with 45 CFR 164.514(a) and (b) (Code of Federal Regulations Title 45, Public Welfare) and therefore independent review board approval was not required.

#### Study variables

##### Clinical characteristics

Charlson Comorbidity Index (CCI) scores during the 12 months prior to index PCa diagnosis were measured. PCa-related characteristics were captured at the time of PCa diagnosis including tumor grade, Gleason score, and risk category based on the D’Amico risk classification. The index PSA was defined as the last PSA value before or on the index biopsy date.

##### Monitoring patterns

Monitoring patterns included PSA test, biopsy, and urology visits.

##### Overall survival

Overall survival was estimated per 100 person-years (PY).

#### Statistical methods

Clinical characteristics were examined descriptively and compared between the OBS and IMT cohorts. Chi-square and t-tests were used to calculate *p*-values, respectively, for categorical and continuous variables. Logistic regression was used to determine odds ratios and 95% confidence intervals (CIs) of factors associated with the selection of OBS versus IMT. Based on model fitting and clinical rationale, covariates adjusted in the model included age, race, marital status, insurance status, family history of PCa, prior diagnosis of chronic obstructive pulmonary disease, CCI score, and D’Amico risk categories. The use and frequency of monitoring patterns were assessed for patients utilizing OBS before switching to active treatment during the first, second, and third years following their index PCa diagnosis.

Unadjusted Kaplan-Meier (KM) and log rank tests were used to compare survival rates in OBS and IMT cohorts. Multivariate Cox proportional hazards models were used to examine Hazard Ratio (HR), and 95% CI of overall survival.

Statistical analyses were performed using SAS Version 9.3 (Cary, NC) with *p*-value < 0.05 considered significant. Standardized differences and clinical relevance were also considered.

#### Subgroup analyses

A subgroup analysis was conducted by reporting monitoring patterns among men whose treatment was managed with OBS: 1) for those who remained on OBS for at least 18 months and had ≥1 urologist visit (a proxy for active patient management), and 2) for those who remained on OBS for ≥24 months.

#### Chart review

Manual examination of de-identified EMR chart data was undertaken to explore why some OBS patients did not have follow-up visits. Charts were reviewed for all OBS patients without a follow-up visit in the urology department within the first year after PCa diagnosis (*N* = 57). The reasons were categorized as follow-up by another clinical department (eg, oncology), delayed treatment based on patients’ preference, complicating comorbidities, and limited follow-up data.

## Results

### Patient characteristics

**Fig. 1:**
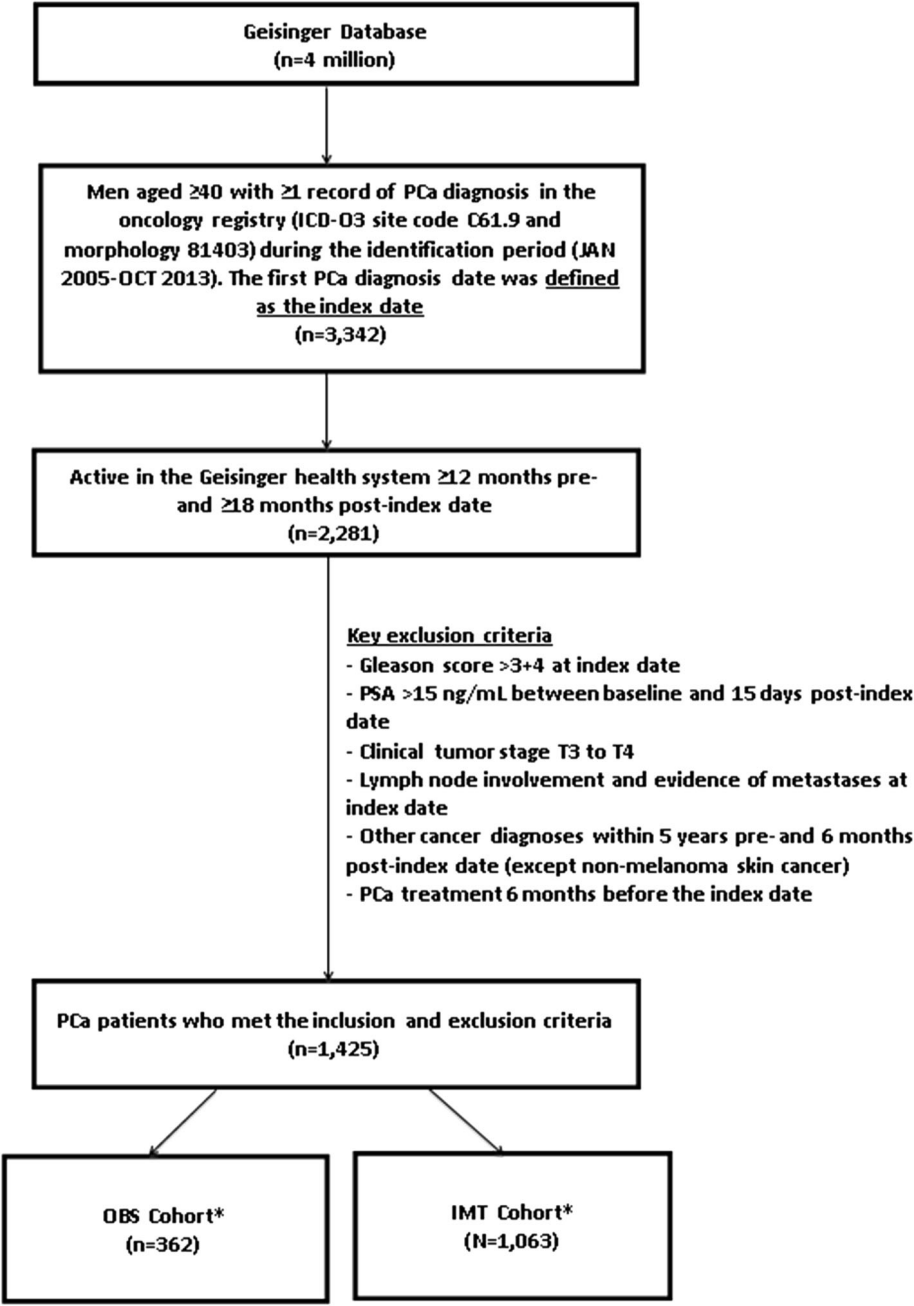
Patient Selection Flow Chart. PSA: prostate specific antigen; PCa: prostate cancer; ICD: international classification of diseases; OBS: observation strategies; IMT: immediate treatment. *Cohorts were defined as receiving any treatment (IMT) or no treatment (OBS) within 6 months after index PCa diagnosis

A total of 3342 men diagnosed with PCa and aged ≥40 years were identified from the oncology registry. After applying the exclusion criteria, the remaining 1425 men in this analysis included 362 patients (25.5%) in the OBS cohort vs 1063 patients (74.5%) in the IMT cohort (Fig. 1) with a median follow-up of 5 years.

There were no significant differences between the OBS and IMT groups in index PSA result, tumor stage, or body mass index (Tables 1 and 2). The OBS group was older (65.6 vs 62.8 years; *p* < 0.01), had higher CCI scores (CCI score ≥ 2: 21.5% vs 12.2%, *p* < 0.01), more patients whose marital status was “divorced” (9.1% vs 5.7%; *p* = 0.03), fewer patients whose marital status was “married” (72.9% vs 80.9%; *p* < 0.01) and more Medicare beneficiaries (32.3% vs 24.2%; *p* < 0.01) (Table 1). The OBS cohort also had a lower proportion with Gleason score = 3 + 4 at diagnosis (22.9% vs 35.5%, *p* < 0.01), and a higher proportion of low-risk PCa (65.2% vs 55.0%, *p* < 0.01) (Table 2).

**Table 1:** Socio-demographic characteristics of men with favorable risk PCa in OBS vs IMT cohorts

	OBS		IMT			
	(*n* = 362)		(*n* = 1063)			
	N/Mean	%/SD	N/Mean	%/SD	*P*-value	Standardized difference ^a^
Age (Mean)	65.6	9	62.8	8	<.01	33.10
Age Group						
40–64	166	45.9%	601	56.5%	<.01	21.47
65–74	137	37.9%	390	36.7%	0.69	2.39
75+	59	16.3%	72	6.8%	<.01	21.47
Race						
White	347	95.9%	1044	98.2%	0.01	13.91
Black or African American	14	3.9%	11	1.0%	<.01	18.37
Native Hawaiian	1	0.3%	4	0.4%	0.78	1.75
Asian	0	0.0%	1	0.1%	0.56	4.34
Unknown	0	0.0%	3	0.3%	0.31	7.52
Marital Status						
Single	33	9.1%	68	6.4%	0.08	10.17
Married	264	72.9%	860	80.9%	<.01	18.99
Widow	30	8.3%	67	6.3%	0.20	7.63
Divorced	33	9.1%	61	5.7%	0.03	12.89
Separated	2	0.6%	7	0.7%	0.83	1.37
Insurance Status						
Medicare	117	32.3%	257	24.2%	<.01	18.15
Veterans Affairs	2	0.6%	2	0.2%	0.26	5.99
Medicaid	8	2.2%	19	1.8%	0.61	3.02
Commercial	227	62.7%	782	73.6%	<.01	23.44
Other	4	1.1%	3	0.3%	0.05	9.91
Unknown	4	1.1%	0	0.0%	<.01	14.93

*OBS* observation, *IMT* immediate treatment, *SD* standard deviation, *CCI* Charlson Comorbidity Index

^a^SD = standardized difference (SD is defined as the difference in sample means or proportions divided by standard error; reported as 100*|actual standardize difference|. Standardize differences >|10| are considered significant

**Table 2:** Clinical characteristics of men with favorable risk PCa in OBS vs IMT cohorts

	OBS		IMT			
	(*n* = 362)		(*n* = 1063)			
	N	%	N	%	*p*-value	Standardized difference ^a^
Comorbid Indices (CCI)						
0	225	62.2%	750	70.6%	<.01	17.83
1	59	16.3%	183	17.2%	0.69	2.45
2+	78	21.6%	130	12.2%	<.01	25.04
Comorbid Conditions						
Hypertension	153	42.3%	405	38.1%	0.16	8.50
Diabetes	61	16.9%	134	12.6%	0.04	11.99
COPD	43	11.9%	82	7.7%	0.02	14.03
Congestive Heart Failure	13	3.6%	27	2.5%	0.30	6.09
Dementia	0	0.0%	3	0.3%	0.31	7.52
Benign Prostatic Hyperplasia	130	35.9%	388	36.5%	0.84	1.22
Body Mass Index						
Underweight (< 18.5)	1	0.3%	5	0.5%	0.62	3.18
Normal (18.5–24.9)	42	11.6%	102	9.6%	0.27	6.52
Overweight (25.0–29.9)	118	32.6%	342	32.2%	0.88	0.90
Obese (≥30)	112	30.9%	336	31.6%	0.81	1.44
Unknown	89	24.6%	278	26.2%	0.56	3.60
Family History of Prostate Cancer						
Yes	74	20.4%	262	24.7%	0.10	10.07
No	208	57.5%	598	56.3%	0.69	2.43
Unknown	80	22.1%	203	19.1%	0.22	7.42
Family History of Cancer	109	30.1%	339	31.9%	0.53	3.85
Prostate Cancer Characteristics						
Index PSA (Mean, SD)	5.8	2.5	5.7	2.5	0.31	6.31
< 4 ng/mL	69	19.1%	208	19.6%	0.83	1.28
4–10 ng/mL	256	70.7%	758	71.3%	0.83	1.30
> 10 ng/mL	30	8.3%	77	7.2%	0.52	3.90
Unknown	7	1.9%	20	1.9%	0.95	0.38
Index Clinical Stage ^b^						
Stage 1	303	83.7%	855	80.4%	0.17	8.52
Stage 2	55	15.2%	184	17.3%	0.35	5.73
Unknown	4	1.1%	24	2.3%	0.17	8.97
Index Total Gleason Score = 3 + 4	83	22.9%	377	35.5%	<.01	27.82
Risk Category ^c^						
Low-risk	236	65.2%	585	55.0%	<.01	20.84
Intermediate-risk	117	32.3%	461	43.4%	<.01	22.91
Unknown	9	2.5%	17	1.6%	0.28	6.27

*OBS* observation, *IMT* immediate treatment, *SD* standard deviation, *COPD* chronic obstructive pulmonary disease

^a^SD = standardized difference (SD is defined as the difference in sample means or proportions divided by standard error; reported as 100*|actual standardize difference|. Standardize differences >|10| are considered significant

^b^Clinical stage: anatomic Extent of the disease based on the clinical T, N and M element

^c^Risk Categories: Low risk (T1-T2A, PSA level ≤ 10 ng/mL, and Gleason score ≤ 6) and intermediate-risk (T1 or T2, PSA level > 10 and ≤ 20 ng/mL, and Gleason score = 7)

### Factors associated with the selection of OBS vs IMT

**Fig. 2:**
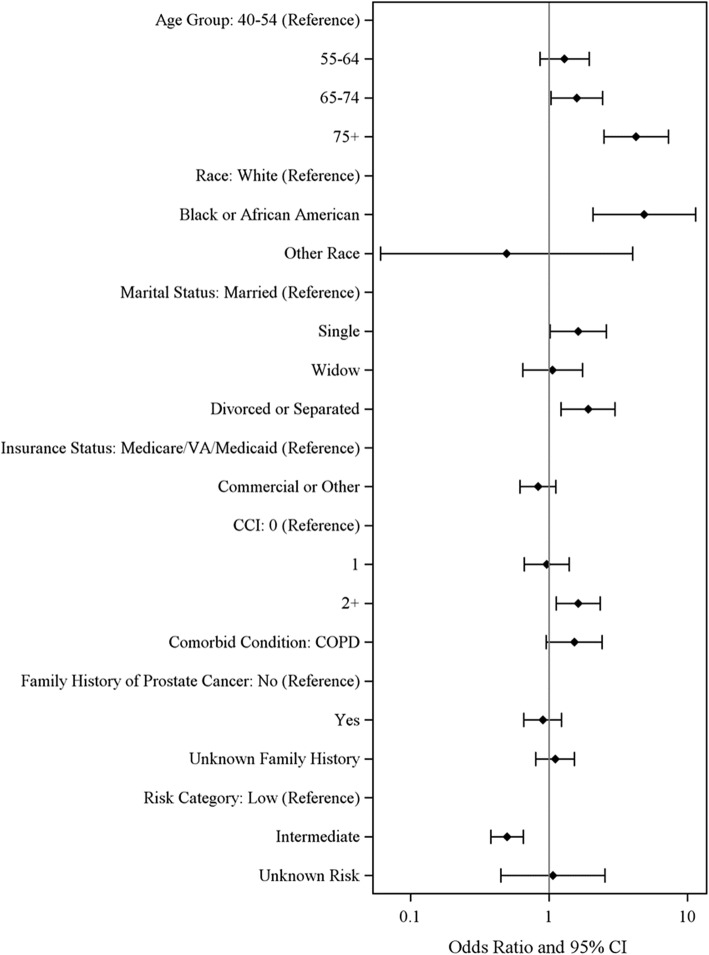
Logistic Regression: Risk Factors Associated with OBS vs IMT. CCI: Charlson comorbidity index; CI: confidence interval; COPD: chronic obstructive pulmonary disease; VA: veterans affairs

Figure 2 shows the factors associated with OBS vs IMT using a logistic regression model. Older age (65–74 years; OR = 1.6 or 75+ years; OR = 4.3), marital status (single; OR = 1.6 or divorce/separated; OR = 1.9), high CCI score (≥2; OR = 1.6), and low-risk PCa (intermediate-risk vs low-risk, OR = 0.5) were significant predictors of choosing OBS over IMT, after adjusting for other covariates.

### Prostate Cancer management trends

**Fig. 3:**
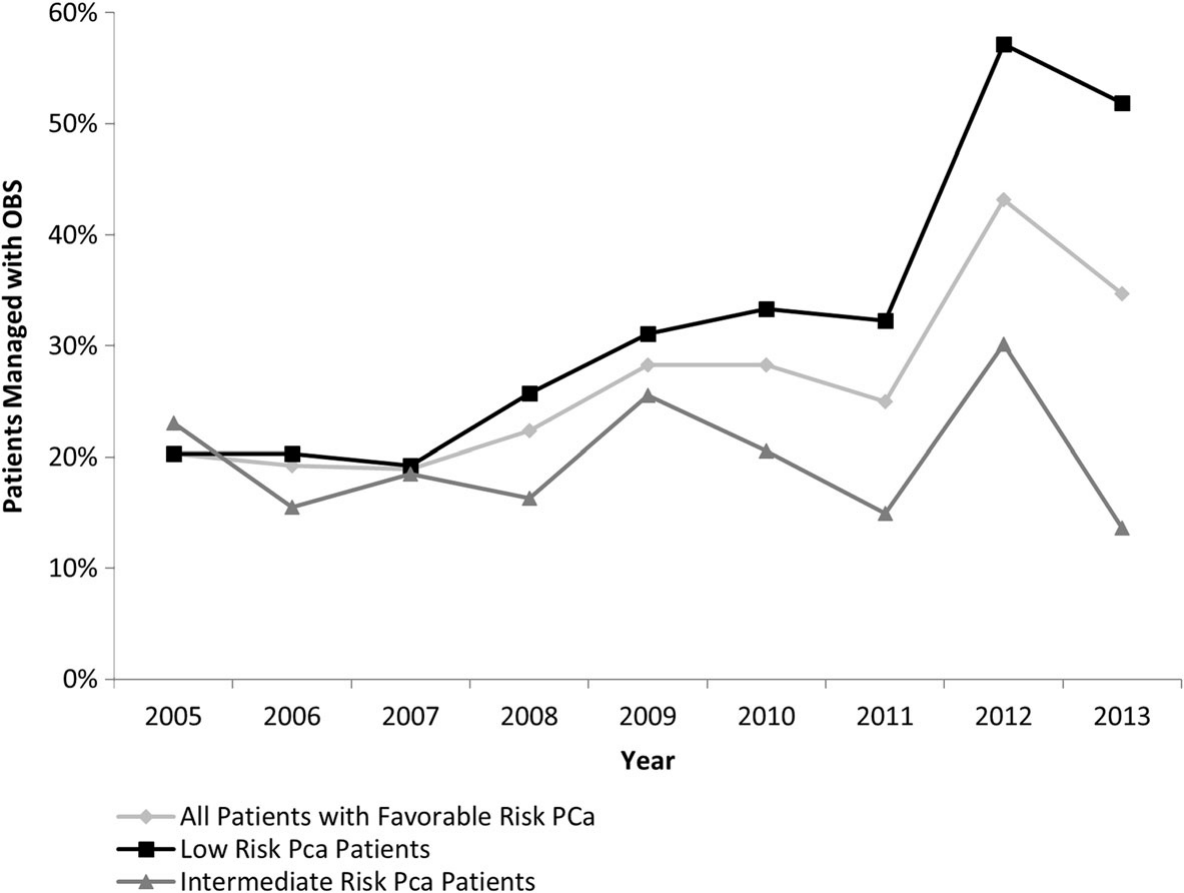
Annual Prostate Cancer Management Trends for Favorable Risk and Low Risk PCa Patients. PCa: prostate cancer; OBS: observation strategies

The proportion of men managed with OBS increased from 20% in 2005 to 35 and 52% in 2013 for patients with favorable risk PCa, or low-risk PCa, respectively (Fig. 3). A similar increase was observed in patients with intermediate-risk PCa up to 2012 (from 19% in 2005 to 30% in 2012) but the proportion decreased to 14% in 2013 (Fig. 3).

### Time to active treatment

**Fig. 4:**
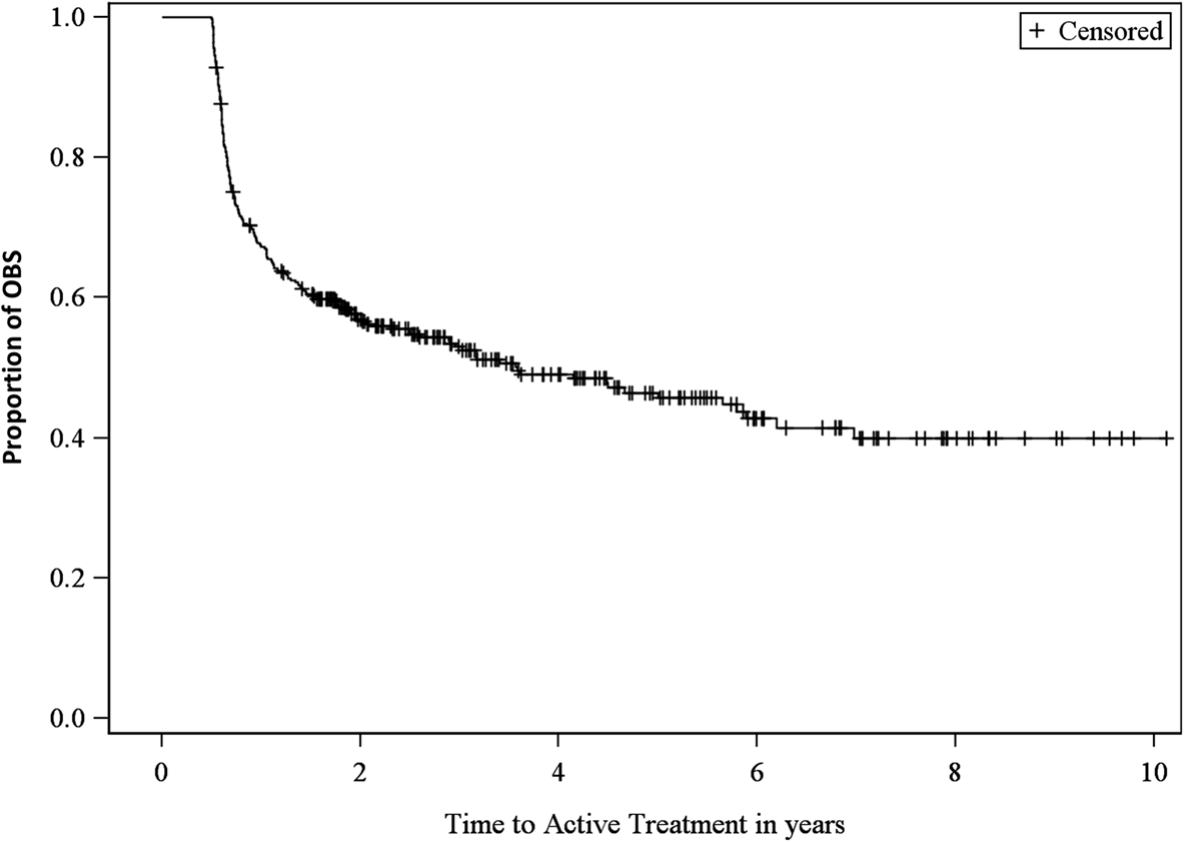
Kaplan Meier for Time to Active Treatment in Men with Favorable Risk PCa Managed with OBS. OBS: observation strategies

The median time to treatment of all OBS patients was ~ 4 years (Fig. 4). Of 181 OBS patients who switched to active treatment, the average time from initial PCa diagnosis to treatment was 459 days (1.3 years).

For the subgroup analysis according to D’Amico risk classification: 29% (236/821) of low-risk versus 20% (117/578) of intermediate-risk PCa patients received OBS. Among men managed by OBS, 50% of low-risk versus 53% of intermediate-risk PCa patients eventually received treatment during the follow-up period.

### Monitoring patterns for men managed with OBS

**Fig. 5:**
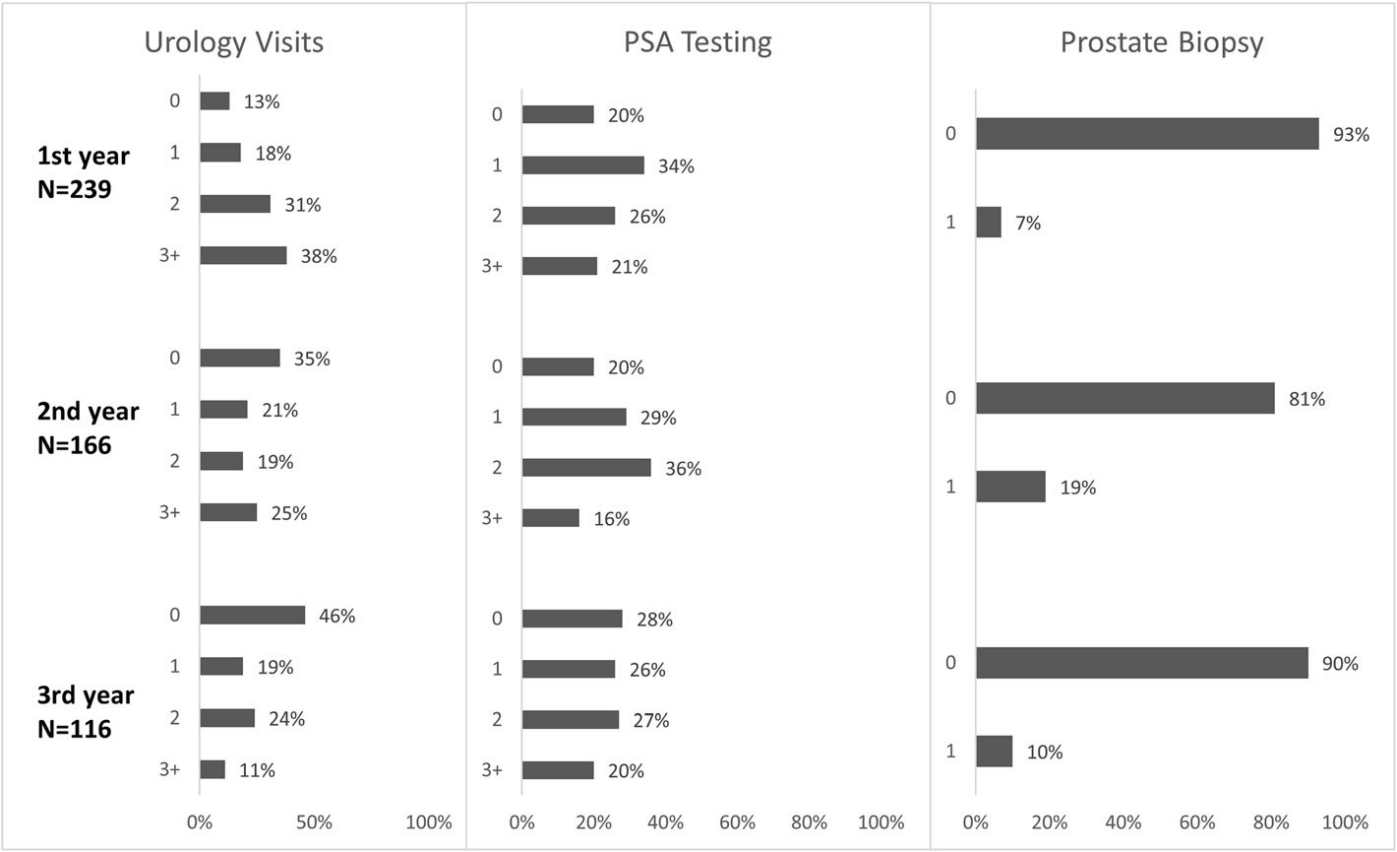
Monitoring Patterns for PCa Patients Managed with OBS. PSA: prostate specific antigen

Monitoring patterns were assessed among the patients remaining on OBS in year 1 (*n* = 239), year 2 (*n* = 166) and year 3 (*n* = 116) (Fig. 5). In the OBS cohort, the percentage of patients with at least one urology visit decreased during the first 3 years post-index PCa diagnosis from 86.6% in year 1 to 65.1% in year 2 and 54.3% in year 3. However, PSA testing rates remained similar in the first 3 years of diagnosis, with approximately 70% of men receiving ≥1 PSA test each year. Approximately 7.1, 18.7, and 10.3% of men in the OBS cohort received a prostate biopsy in the first, second, and third year, respectively (Fig. 5).

Among men remaining on OBS for ≥18 months and with ≥1 urology visit (*N* = 212), 85.8% received ≥1 PSA tests and 19.3% were administered one biopsy within the 18 months. Among those remaining on OBS for ≥24 months (*N* = 166), 88.6% received ≥1 PSA tests and 26.5% received one biopsy within the 24 months. A chart review of 57 patients in the OBS cohort without follow-up urology visits within the first 360 days post-diagnosis indicated that in 49 of 57 cases (86%) of PCa-related care by other GHS departments were identified with oncology as the most common specialty.

### Overall survival

**Fig. 6:**
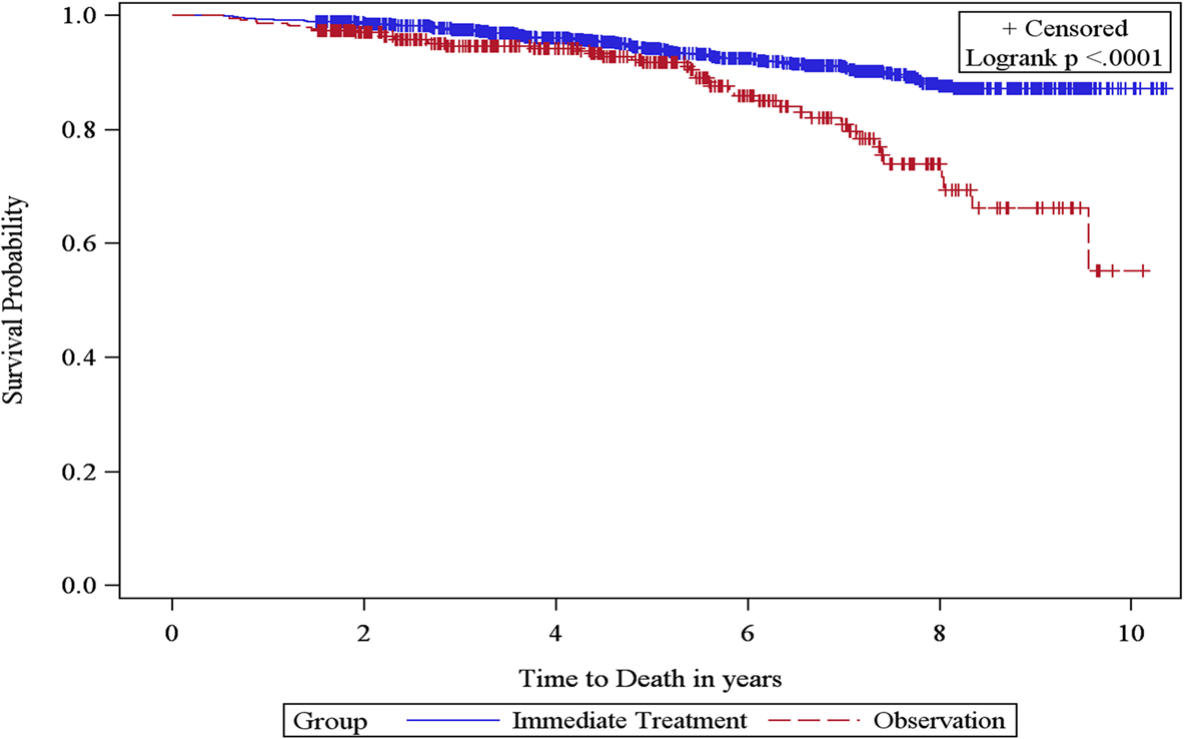
Kaplan Meier Curve for Overall Survival Among Men With Favorable Risk PCa

**Fig. 7:**
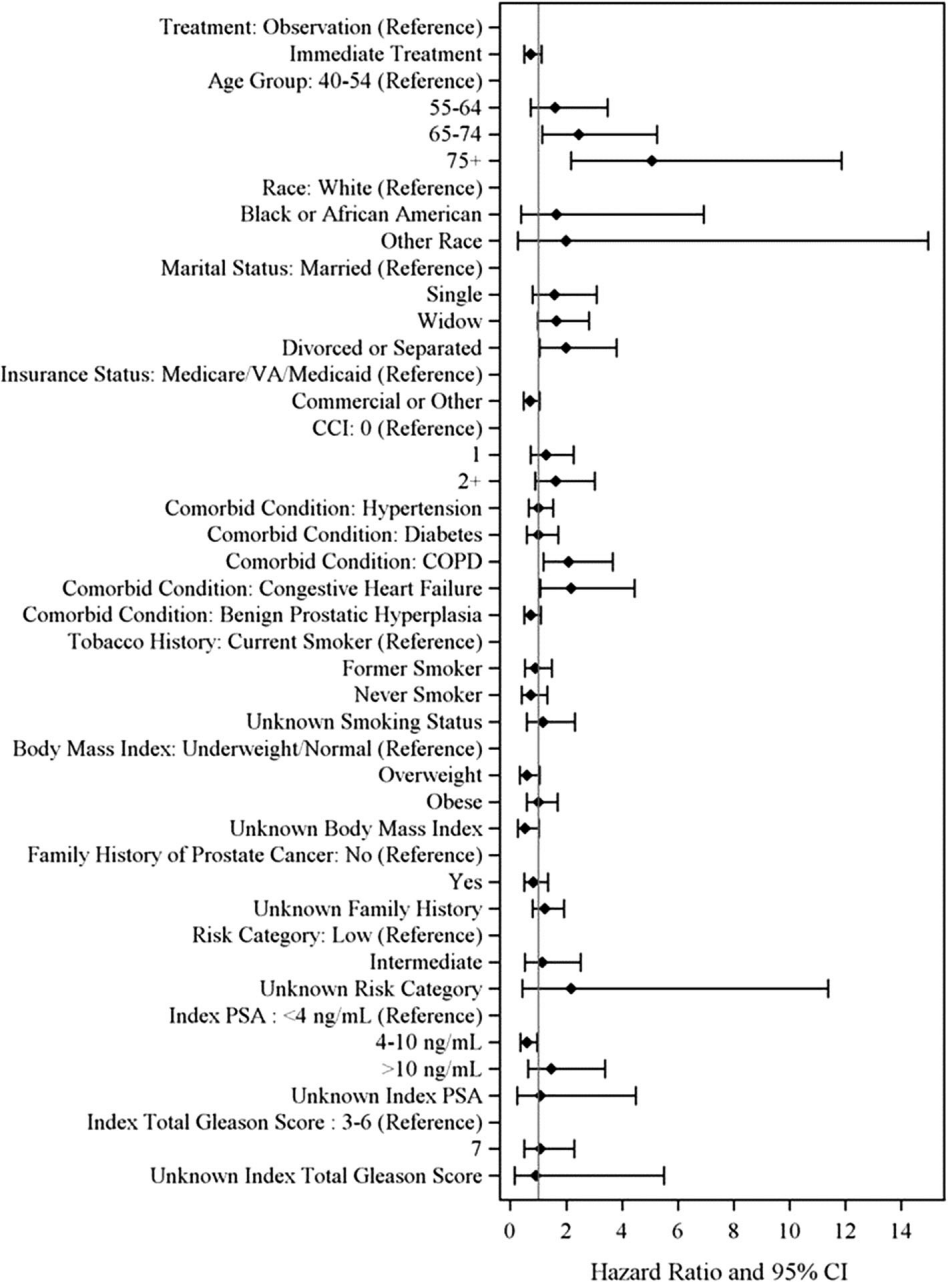
Multivariate Cox Regression Model for All-cause Mortality Among. Men With Favorable Risk PCa. PCa: prostate cancer; VA: veterans affairs; CCI: Charlson comorbidity index; COPD: chronic obstructive pulmonary disorder; PSA: prostate specific antigen; CI: confidence interval

Median follow-up time from PCa diagnosis in the entire study cohort was approximately 5 years. The unadjusted all-cause mortality rate was higher for OBS compared with IMT (2.7 vs 1.3/100 PY; *p* < 0.001). The cumulative hazard plot for overall survival indicated that after the sixth year of PCa diagnosis, the risk of all-cause mortality was higher in the OBS cohort than in the IMT cohort (Fig. 6). However, after multivariate adjustment, there was no significant difference in all-cause mortality between the OBS and IMT cohorts (IMT vs OBS, HR = 0.73, *p* = 0.138; Fig. 7). Multivariate analysis also indicated that older age, divorce or separated status; prior diagnosis of COPD, and congestive heart failure were risk factors for all-cause mortality.

## Discussion

In this study, we described overall survival and the characteristics and management patterns in men with favorable risk PCa within a large community-based health care system in the United States. While no single source provides comprehensive data for most US patients, the major strength of this study is its large community-based setting. In addition to the real-world use of EMR, the claims oncology registry and unstructured information from charts maximized the available data for each patient, while also yielding a large sample size. Each of the data sources provided complementary information.

In the current analysis, we found that patients who were managed by OBS strategies were on average over age 65 years and had higher CCI scores when compared to patients who received definitive treatment after index PCa diagnosis. Factors associated with the selection of OBS were similar with Liu et al. [11]. Similarly, prior research has reported that OBS strategies focus on deferring PCa treatment in older, sicker patients diagnosed with a prostate tumor that is less aggressive than their underlying comorbidities [10, 12].

Because of these known demographic and clinical differences in patients who were managed by OBS compared to those who underwent IMT, the unadjusted all-cause mortality rate was higher in OBS patients compared to IMT. These findings are similar to a previously published study comparing radical prostatectomy with WW in early PCa patients [13]. After adjusting for these known differences in baseline demographic and clinical characteristics between the OBS and IMT cohorts in a multivariate analysis, there was no longer a significant difference in all-cause mortality between the OBS and IMT cohorts. However, longer follow-up is needed to more convincingly assess all-cause mortality. We also evaluated deaths due to PCA or complication from PCa. However, the results were not conclusive because we could not identify the cause of death with certainty. There were a total of nine patients (2.5%) with deaths possibly or probably due to PCa in the OBS group vs 15 deaths (1.4%) possibly or probably due to PCa in the IMT group (results not shown). In addition, PCa-specific mortality in favorable risk cancer is relatively low and we do not have sufficient numbers of patients followed for a long enough period to appreciate the differences between the OBS and IMT groups.

Other studies have shown that the presence of significant others in a patient’s life often influences treatment decisions and our results suggest that even adjusting for other factors such as race and type of insurance, that men who were divorced or separated (or single) were more likely to be treated with OBS vs IMT. However, in this study, marital status was only captured as single, married, widow, divorced, and separated, and data on whether men who were divorced, separated, or single were actually living alone or living with a significant other was not captured [14]. In addition, health insurance plans were identified from the data including Medicare, commercial, Medicaid, self/other, Veterans Affairs, and unknown. We observed from our study that the risk of choosing OBS was significantly lower among the patients enrolled in commercial/other types of health insurance plans (34.5%) compared to those enrolled in Medicare/VA/Medicaid health insurance plans.

Regarding the adoption of OBS, we found an increased use of OBS in the GHS over time. This trend was consistent with American Urological Association (AUA) guideline changes and was similar with the trend in the adoption of AS/WW from a large US national registry population with low-risk PCa [15]. Our study also indicated that patients who switched from OBS to IMT (64.6 years) were, on average, younger than patients who remained in the OBS (66.3 years) cohort with a median follow-up period of 5 years. Moreover, 50% of the men on OBS received active treatment eventually which is higher than in other studies [16–20]. This might be because the benefits of AS were not clearly discussed or due to lesser adoption of AS in community settings as opposed to academic institutions. This percentage is plausible given that during the timeframe of the study (January 2004 to April 2015), there were no existing AS guidelines to inform decisions on conversion of patients to active treatment.

The proportion of OBS patients converting to treatment within the first 12 months and the second 12 months after the index date is substantial. The most likely reason for the large drop during the first 12 months is patients simply deferred treatment. A chart review of 20 randomly-selected OBS patients showed that 10 of 20 (50%) patients were considered for IMT. One additional patient received brachytherapy 10 months after diagnosis, but there were limited encounters with urology; therefore, details of the complete treatment plan were not available. Eight of these 11 men (73%) had intentional delays in their treatment date due to comorbid medical conditions to be addressed prior to surgery or to better align with personal commitments such as a seasonal work schedule.

Several AS schedules for the management of favorable risk PCa have been used in various studies including the 2017 National Comprehensive Cancer Network NCCN guideline [21] and Cancer Care Ontario’s guideline on AS for the management of favorable risk PCa endorsed by the American Society of Clinical Oncology (ASCO) [22]. In comparison to these guidelines, we found that serial monitoring in GHS was relatively low.

Among the OBS patients who were active in the GHS for ≥2 years and who remained on OBS treatment in the first 2 years, only 61.5% had 3 or more PSA tests and only 26.5% had a prostate biopsy, which differs from ASCO guidelines and most prospective AS protocols. Our data also showed a relatively stable PSA testing rate but a decrease in urology visits over time. In our study, 7.7, 20.5, and 10.8% of patients had one prostate biopsy in the first, second, and third year, respectively. Magnetic resonance imaging (MRI) use is an increasingly important tool in clinical practice for the diagnosis and monitoring of PCa patients, especially the multi-parametric (mp) MRI [23]. Using Current Procedural Terminology (CPT) codes to identify MRI use during the study period, we observed that only 8.56% of the patients who were in the OBS group and 8.75% of the patients with IMT had prostate-related MRI during the follow-up period (results not shown). It is worth mentioning that MRI, including mpMRI, was not routinely used during the timeframe of this study (January 2004 to April 2015). Although MRI was not routinely carried out in the management of PCa in these patients, we do not believe that any of these patients had their cancer management affected by not using mpMRI.

The low monitoring rates observed in this study may reflect a greater proportion of WW versus AS, which we are unable to distinguish in our OBS population. In addition, a large proportion of patients included in this analysis were managed or treated prior to 2010 when AS was not yet widely adopted as a management strategy for patients with clinically-localized PCa and there were no consensus/guidelines for monitoring of AS or WW. Low monitoring rates for AS and WW in the community setting have been reported by Herden and Weissbach [24] and Auffenberg et al. [25] as well, suggesting a need for research into the factors associated with departure from guideline-recommended monitoring practices.

This study was a retrospective review of community practice intended to reflect the real-world implementation of routine management. The inclusion and exclusion criteria were primarily based on the patient having a lower-risk, early stage of cancer at diagnosis. To achieve the observational nature of the study, no requirements or exclusions were made based upon management protocol including the use of imaging data or biopsy results to address diagnosis or treatment among favorable risk PCa patients.

In sum, our data reveal significant opportunities for improvement in management strategies for favorable risk prostate cancer within our large community group setting. Educational activities should promote increased adoption of AS among favorable risk PCa patients as well as a clear distinction between AS and WW in individual patients. Community-based AS strategies should also focus on appropriate monitoring of patients placed on AS.

## Limitations

All patients without definitive treatment in the first 6 months after PCa diagnosis were temporarily classified as receiving OBS. However, the OBS strategies, AS or WW, were not prospectively collected or always clearly noted by practitioners in the patient medical records. Therefore, we were not able to clearly distinguish between AS or WW from these real-world data. This limitation was also noted from other studies (Cooperberg et al., 2015) [15]. Furthermore, there also was some apparent misclassification in assigning patients to OBS strategies versus IMT by the 6-month threshold, as some patients planned for definitive treatment but waited longer than 6 months before receiving treatment. An additional limitation is that Geisinger serves patients in Central and Northeastern Pennsylvania; therefore, the study results may not be generalizable to the entire US PCa population. Finally, some patients may have received care outside of GHS.

## Conclusion

The proportion of men managed by OBS increased over the 10-year study period although the rate of adoption might have been slightly slower in this large community setting compared with prospective AS protocols. Men in the OBS cohort had a higher proportion of low-risk PCa but were older and had higher CCI scores. However, no differences were observed in overall survival between the two groups after covariates adjustment. These data provide insights into management patterns for favorable risk PCa within a community setting.

Analysis of monitoring patterns within the OBS cohort showed relatively low rates of repeat clinic visits and testing. Further research is necessary to focus on verifying the appropriate monitoring schedule to optimize patient outcomes and to encourage the adoption of AS in community settings.

## Abbreviations

AS: Active surveillance
ASCO: American Society of Clinical Oncology
CCI: Charlson comorbidity index
CPT: Current procedural terminology
EMR: Electronic medical records
GHS: Geisinger Health System
IMT: Immediate treatment
mpMRI: Multi-Parametric magnetic resonance imaging
MRI: Magnetic resonance imaging
OBS: Observation strategies
PSA: Prostate specific antigen
PY: person years
WW: Watchful waiting

## References

[1] Polascik TJ, Passoni NM, Villers A, Choyke PL (2014). Modernizing the diagnostic and decision-making pathway for prostate cancer. Clin Cancer Res.

[2] Schröder FH, Hugosson J, Roobol MJ, Tammela TL, Ciatto S, Nelen V (2012). Prostate-cancer mortality at 11 years of follow-up. N Engl J Med.

[3] Roobol MJ, Kerkhof M, Schröder FH, Cuzick J, Sasieni P, Hakama M (2009). Prostate cancer mortality reduction by prostate-specific antigen-based screening adjusted for nonattendance and contamination in the European randomised study of screening for prostate Cancer (ERSPC). Eur Urol.

[4] Mottet N, Bellmunt J, Bolla M, Joniau S, Mason M, Matveev V (2011). EAU guidelines on prostate cancer. Part II: treatment of advanced, relapsing, and castration-resistant prostate cancer. Actas Urol Esp.

[5] Eldefrawy A, Katkoori D, Abramowitz M, Soloway MS, Manoharan M (2013). Active surveillance vs. treatment for low-risk prostate cancer: a cost comparison. Urol Oncol.

[6] Montironi R, Hammond EH, Lin DW, Gore JL, Srigley JR, Samaratunga H (2014). Consensus statement with recommendations on active surveillance inclusion criteria and definition of progression in men with localized prostate cancer: the critical role of the pathologist. Virchows Arch.

[7] Ruseckaite R, Beckmann K, O’Callaghan M, Roder D, Moretti K, Millar J (2016). A retrospective analysis of Victorian and South Australian clinical registries for prostate cancer: trends in clinical presentation and management of the disease. BMC Cancer.

[8] Barocas DA, Cowan JE, Smith JA, Carroll PR, Investigators CPSURE (2008). What percentage of patients with newly diagnosed carcinoma of the prostate are candidates for surveillance? An analysis of the CaPSURE™ database. J Urol.

[9] D'Amico AV, Whittington R, Malkowicz SB, Schultz D, Blank K, Broderick GA (1998). Biochemical outcome after radical prostatectomy, external beam radiation therapy, or interstitial radiation therapy for clinically localized prostate cancer. JAMA.

[10] Filson CP, Marks LS, Litwin MS (2015). Expectant management for men with early stage prostate cancer. CA Cancer J Clin.

[11] Liu J, Womble PR, Merdan S, Miller DC, Montie JE, Denton BT (2015). Factors influencing selection of active surveillance for localized prostate Cancer. Urology.

[12] Albertsen PC, Hanley JA, Gleason DF, Barry MJ (1998). Competing risk analysis of men aged 55 to 74 years at diagnosis managed conservatively for clinically localized prostate cancer. JAMA.

[13] Holmberg L, Bill-Axelson A, Helgesen F, Salo JO, Folmerz P, Haggman M (2002). A randomized trial comparing radical prostatectomy with watchful waiting in early prostate cancer. N Engl J Med.

[14] Allen JD, Akinyemi IC, Reich A, Fleary S, Tendulkar S, Lamour N. African American women's involvement in promoting informed decision-making for prostate cancer screening among their partners/spouses. Am J Mens Health. 2018; 10.1177/1557988317742257. 10.1177/1557988317742257 PMC6131450 29298558

[15] Cooperberg MR, Carroll PR (2015). Trends in management for patients with localized prostate cancer, 1990-2013. JAMA.

[16] Newcomb LF, Thompson IM, Boyer HD, Brooks JD, Carroll PR, Cooperberg MR (2016). Outcomes of active surveillance for clinically localized prostate cancer in the prospective, multi-institutional Canary PASS cohort. J Urol.

[17] Cooperberg MR, Cowan JE, Hilton JF, Reese AC, Zaid HB, Porten SP (2010). Outcomes of active surveillance for men with intermediate-risk prostate caner. J Clin Oncol.

[18] Lang MF, Tyson MD, Alvarez JR, Koyama T, Hoffman KE, Resnick MJ (2017). The influence of psychosocial constructs on the adherence to active surveillance for localized prostate cancer in a prospective, population-based cohort. Urology.

[19] Loeb S, Folkvaljon Y, Makarov DV, Bratt O, Bill-Axelson A, Stattin P (2015). Five-year nationwide follow-up study of active surveillance for prostate cancer. Eur Urol.

[20] Aizer AA, Gu X, Choueiri TK, Martin NE, Efstathiou JA, Hyatt AS (2015). Cost implications and complications of overtreatment of low-risk prostate cancer in the United States. J Natl Compr Cancer Netw.

[21] Mohler JL, Lee RJ, Antonarakis ES, Armstrong AJ, D-Amico AV, Davis BJ, et al. NCCN clinical practice guidelines in oncology (NCCN Guidelines): Prostate Cancer, Version 2.2018. National Comprehensive Cancer Network. 2018. https://www.nccn.org/professionals/physician_gls/pdf/prostate.pdf. Accessed 31 May 2018

[22] Chen RC, Rumble RB, Loblaw DA, Finelli A, Ehdaie B, Cooperberg MR (2016). Active surveillance for the management of localized prostate cancer (Cancer Care Ontario guideline): American Society of Clinical Oncology clinical practice guideline endorsement. J Clin Oncol.

[23] Ahmed HU, El-Shater Bosaily A, Brown LC, Gabe R, Kaplan R, Parmar MK (2017). Diagnostic accuracy of multi-parametric MRI and TRUS biopsy in prostate cancer (PROMIS): a paired validating confirmatory study. Lancet.

[24] Herden J, Weissbach L. Utilization of active surveillance and watchful waiting for localized prostate cancer in the daily practice. World J Urol. 2018;36(3):383–91. 10.1007/s00345-018-2175-0 29330583

[25] Auffenberg G, Luckenbaugh A, Hawken S, Dhir A, Linsell A, Kaul S, et al. MP25-12 Analysis fo active surveillance follow-up: how closely are patients monitored over time? J Urol. 2016;195(4):e283–4.

